# Exposure to air pollution and the risk of type II diabetes mellitus: a time-series study

**DOI:** 10.3389/fendo.2024.1482063

**Published:** 2024-12-03

**Authors:** Zhuomin Hou, Yongbin Wang, Zhigang Chen, Siyu Sun, Na Xie, Yingen Chen, Lujie Wang, Fei Lin, Guoan Zhao

**Affiliations:** ^1^ Department of Cardiology, The First Affiliated Hospital of Xinxiang Medical University, Weihui, China; ^2^ Department of Epidemiology and Health Statistics, School of Public Health, Xinxiang Medical University, Xinxiang, China; ^3^ Life Science Research Center, The First Affiliated Hospital of Xinxiang Medical University, Weihui, China; ^4^ The Cardiology Department of the Third Affiliated Hospital of Xinxiang Medical University, Xinxiang, China

**Keywords:** air pollution, type 2 diabetes mellitus, hospital costs, length of stay, season, timeseries study

## Abstract

**Background:**

Environmental factors have been identified as primary risk factors for type 2 diabetes mellitus (T2DM). However, studies on the association between environmental factors and T2DM have mainly focused on morbidity and mortality, which do not fully reflect the disease burden stemming from air pollution. Therefore, we aimed to evaluate the correlation between air pollution and T2DM, including hospital length of stay (LOS) and costs.

**Methods:**

We collected data on patients with T2DM from three healthcare institutions in Xinxiang from 2016–2021. Data on particulate and gaseous pollutants in Xinxiang and daily meteorological data were collected from national databases. The distribution lag nonlinear model was used to evaluate the correlation between air pollution and the number of inpatients with T2DM, LOS, and hospital costs. Subgroup analyses were conducted to identify potential modifying factors.

**Results:**

Overall, 13,797 patients with T2DM were included in our analysis. Within the cumulative lag of 7 days, with every increase of 1 mg/m^3^ of carbon monoxide (CO) and 10 μg/m^3^ of 2.5 microns particulate matter, nitrogen dioxide and ozone exhibited significant associations with an increase in diabetes hospitalization risk. CO exhibited adverse effects on LOS on most lag days. Moreover, hospital costs were significantly associated with the attributable fraction of LOS and hospital costs attributed to diabetes.

**Conclusions:**

Exposure to air pollutants increased T2DM risk, imposing significant economic and social burdens in Xinxiang, China. Implementing policies to reduce air pollutant exposure may decrease T2DM admissions, costs, and LOS.

## 1 Introduction

Type 2 diabetes mellitus (T2DM) significantly contributes to the global disease burden and risk of premature death (1). One in eleven adults in the world has diabetes, and 90% of them have T2DM (2). The average time of death in diabetic patients is 6 years earlier than non-diabetic patients, and every 10 years of diabetes diagnosis, life expectancy decreases by about 4 years (3). A global diabetes report published by the World Health Organization stated that the global prevalence of T2DM has risen sharply in the past few decades. Unhealthy lifestyle, obesity, lack of exercise, and malnutrition are widely regarded as risk factors for T2DM (4–6). In addition to these recognized risk factors, environmental impact is increasingly considered as a cause of diabetes (7). Notably, air pollution is the eighth most important factor among the 79 death risk factors (8),and is associated with cardiovascular diseases. In a global and prospective study involving more than 100,000 people in 21 countries, it was found that air pollution was related to the attribution score of 13.9% people with cardiovascular diseases (9). Emerging evidence shows that air pollution is also associated with T2DM (10).

An increasing number of epidemiological studies have identified environmental factors as primary risk factors for T2DM (11–14). More than 20 years have passed since it was found that there is a significant correlation between air pollution and the prevalence of diabetes (15); however, recently this relationship has gained growing attention. A previous meta-analysis demonstrated that exposure to high levels of air pollution is significantly related to the increased prevalence of T2DM (16). 20% of diabetes worldwide is associated with exposure to particulate matter 2.5 microns (PM_2.5_) (17). Moreover, a large cohort study showed that there was a positive correlation between exposure to nitrogen dioxide (NO_2_) and the prevalence of diabetes (18). Furthermore, short-term exposure to PM_2.5_, particulate matter 10 microns (PM_10_), sulfur dioxide (SO_2_), and NO_2_ is positively associated with T2DM mortality (19), and under-diagnosed diabetes risk is associated with long-term ozone (O_3_) pollution in Malaysia (20).

Air pollution prevention measures have been successful worldwide. However, in Henan Province, a study shed light on the severe air pollution situation, especially in the northern part where the majority of the population lives with polluted air (21). Despite China’s efforts to improve air pollution, the health burden caused by air pollution is still high (22). Xinxiang, located in the northern part of Henan, has a population of 6 million and an annual gross domestic product of 300 billion Yuan, making it a critical economically developing region. Xinxiang is a city with relatively developed industries, surrounded by numerous chemical manufacturers and other industrial enterprises (23).

In recent years, increasing attention has been paid to the impact of air pollution on length of stay (LOS) and hospital costs (24). Diabetes not only leads to a reduced standard of living and health risks, but also has a significant economic impact on patients. Labor costs and socio-economic pressures exacerbate the strain on healthcare systems and governments globally (25). Previous studies have primarily focused on morbidity and mortality, which do not fully reflect the disease burden stemming from air pollution. Thus, our analysis aimed to assess the influence of air pollutants on hospital admissions, LOS, and expenses for patients with diabetes in Xinxiang.

## 2 Materials and methods

### 2.1 Patient selection

Data pertaining to patients with diabetes between January 1, 2016 and October 31, 2021 were collected from three healthcare institutions: The First Affiliated Hospital of Xinxiang Medical College, The Third Affiliated Hospital of Xinxiang Medical College, and Xinxiang People’s Hospital. A total of 13,797 patients with diabetes were included. All T2DM cases were clinically diagnosed and validated with the diagnostic code E11 from the 10th edition of the International Classification of Diseases. We recorded data on sex, age (categorized as<65 years old or ≥65 years old), hospital LOS, and hospital costs, which were also included in the database.

This study was approved by the Ethics Committee of the First Affiliated Hospital of Xinxiang Medical College. The ethical batch number is EC-022-140. All data used were collected for administrative purposes without any individual identifiers. Therefore, informed consent was not required.

### 2.2 Air pollutants data

The study included six air pollutants, including both particulate pollutants and gaseous pollutants, with particulate pollutants including PM_2.5_ and PM_10_ and gaseous pollutants including NO_2_, SO_2_, carbon monoxide (CO), and O_3_. Air pollution data for Xinxiang City were collected from the Henan Meteorological Residence for the years 2016–2021. Air pollutant concentrations were 24-h average concentrations. Furthermore, O_3_ concentrations were calculated as the maximum 8-h concentration per day and the daily average. Daily meteorological data for the same period, including minimum, average, and maximum temperatures and relative humidity, were downloaded from the publicly accessible China National Meteorological Data Sharing System (http://data.cma.cn/). Meteorological data were calculated every 24 h.

### 2.3 Statistical analysis

The distribution of air pollutants and meteorological factors were characterized using mean, standard deviation, quartiles, and maximum and minimum values. Poisson’s generalized additive model was used in this study. The confounding factors were the day of the week (DOW), time, and weather. Notably, the incidence of diabetes is affected by the air pollution on the same day and the exposure levels in previous days.

Therefore, we used the distributed lag nonlinear model (DLNM) to study the correlation. Based on the traditional models, such as the generalized linear model and generalized additive model, we used the cross-basis function to transform the characteristics of the variables, which has the following expression:


Log (Yt)=βZt,l+ +ns(time,7)+ns(hum,3)+ns (temp,3)+DOW+holidays,

where Yt represents the number of patients with diabetes hospitalized; Zt denotes the concentration of the six air pollutants at day t; β represents the coefficient for Zt,l, which represents the logarithmic increase in hospitalizations per unit increase in air pollutants; l represents the lag days; ns refers to the restricted cubic spline; df is the degree of freedom (df); and time denotes the time variable (df was 7). The following covariates were applied to the model: df for both humidity and temperature as 3, DOW, and holidays as an indicator of the holiday season.

In the past, the distributed lag linear model (DLM) was widely used to study the health effects of air pollution. However, this model assumes a linear exposure–response relationship. Many exposure–response relationships (such as temperature–death) exhibit a nonlinear relationship, such as U and V, making the DLM unsuitable. To address these problems, in 2006, Armstrong (26) first proposed and applied the DLNM in epidemiology.

In this study, the DLNM was used to analyze the relationship between air pollution and the number of diabetes hospitalizations, days of hospitalization, and hospitalization costs. A 7-day lag was used to assess the lagged and cumulative impacts due to air pollution; it was categorized into single-day and multi-day lag, with the single-day lag ranging from lag 0 to lag 7, and the cumulative lag ranging from lag 01 to lag 07. Subgroup analyses by sex, age, and season were also conducted. Sex was categorized into male and female groups; age was categorized into elderly and non-elderly groups, with a cut-off of 65 years; and seasonal groups were categorized into warm and cold seasons, with May to September as the warm season group and January to February as the cold season group.

We used the forward methodology provided by Gasparrini and Leone (27) to estimate attributable fractions (AFs) and attribution number to quantify the number of days of hospitalization and the cost of hospitalization due to air pollution exposure. We also constructed a two-pollutant model to assess the potential impacts of individual air pollutants. The data outputs were relative risks (RRs) and 95% confidence intervals (CIs) for each 10-unit increase in PM_10_, NO_2_, SO_2_, CO, and O_3_ concentrations, and for each 1-unit increase in CO concentration.

All the calculations were performed using R software version 4.1.0 (R Core Team, Vienna, Austria), with its “DLNM” package. The statistical review of the study was performed by a biomedical statistician.

## 3 Results

### 3.1 Baseline characteristics

This analysis included 13,791 patients with T2DM (**Table 1**). Overall, 137,000 patients with diabetes had a total of 173,200 days of hospital LOS and 96 million Chinese Yuan in hospital costs. The daily ranges for diabetes hospital admissions, LOS, and costs were 0–26, 0–332 days, and 0–263,000 Chinese Yuan, respectively. Among the air pollutants, PM_2.5_ and PM_10_ had the broadest concentration ranges, from 5 μg/m^3^ to 644 μg/m^3^ and 12 μg/m^3^ to 823 μg/m^3^, respectively.

**Table 1:** Distribution characteristics of diabetes, air pollutants, and meteorological factors.

Variables	Mean ± SD	Min	P25	P50	P75	Max
**T2DM cases**	6.47 ± 4.16	0	3	6	9	26
**LOS**	81.28 ± 53.30	0	40	71	115	332
**Hospital cost**	45,249.35 ± 30.40	0	18,827.85	36,604.48	64,247.45	263,620.56
**PM_2.5_**	61.04 ± 47.27	5	32	46	75	644
**PM_10_**	119.69 ± 71.49	12	66	94	136	823
**SO_2_**	21.31 ± 16.88	3	11	16	25	156
**NO_2_**	42.32 ± 19.94	8	27	40	55	168
**CO**	1.15 ± 0.70	0.3	0.7	1	1.37	8
**O_3_**	104.15 ± 50.01	7	60	97	145	276
**Temperature**	16.23 ± 10.11	-8	7.3	17.1	25.5	34.6
**Humidity**	61.26 ± 18.11	13	48	63	75	100

T2DM cases, the number of individuals with type 2 diabetes mellitus; LOS, length of stay; Hospital cost, costs for hospitalization due to type 2 diabetes (Hospital cost is expressed in Chinese Yuan); PM_2.5_, aerodynamic diameter<2.5 mm; PM_10_, aerodynamic diameter<10 mm; SO_2_, sulphur dioxide; NO_2_, nitrogen dioxide; CO, carbon monoxide; O_3_, ozone; Temperature, the temperature in Xinxiang; Humidity, the humidity in Xinxiang; SD, standard deviation.

### 3.2 Data visualization

**Figure 1:**
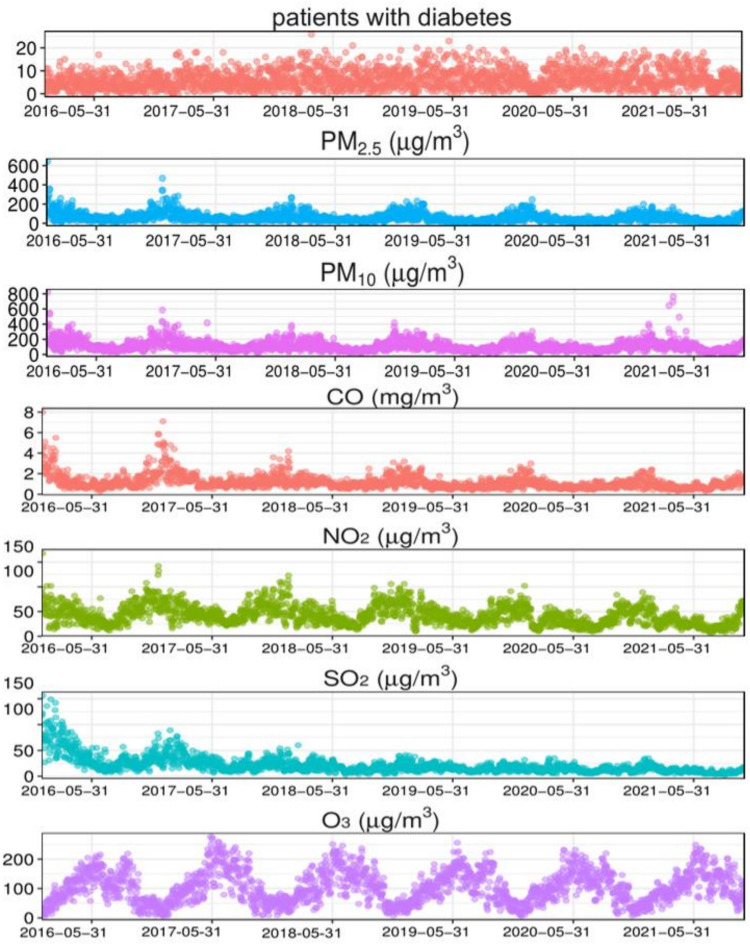
Time-series results regarding the association of diabetes with meteorological factors and air pollution indicators in Xinxiang. PM_2.5_, aerodynamic diameter<2.5 μm; PM_10_, aerodynamic diameter<10 μm; SO_2_, sulfur dioxide; NO_2_, nitrogen dioxide; CO, carbon monoxide; O_3_, ozone.

The time series results of diabetes, the six air pollutants, and the meteorological factors in Xinxiang City from 2016 to 2021 are shown in **Figure 1**. Diabetes hospitalizations showed a fluctuation according to the year; however, the overall trend showed an upward trajectory. The mean temperature and air pollution indicators exhibited significant periodicity and relatively stable patterns. The concentrations of CO, NO_2_, and SO_2_ decreased annually, with higher pollutants concentrations observed during the winter.

### 3.3 Association between pollutants and diabetes admission

**Figure 2:**
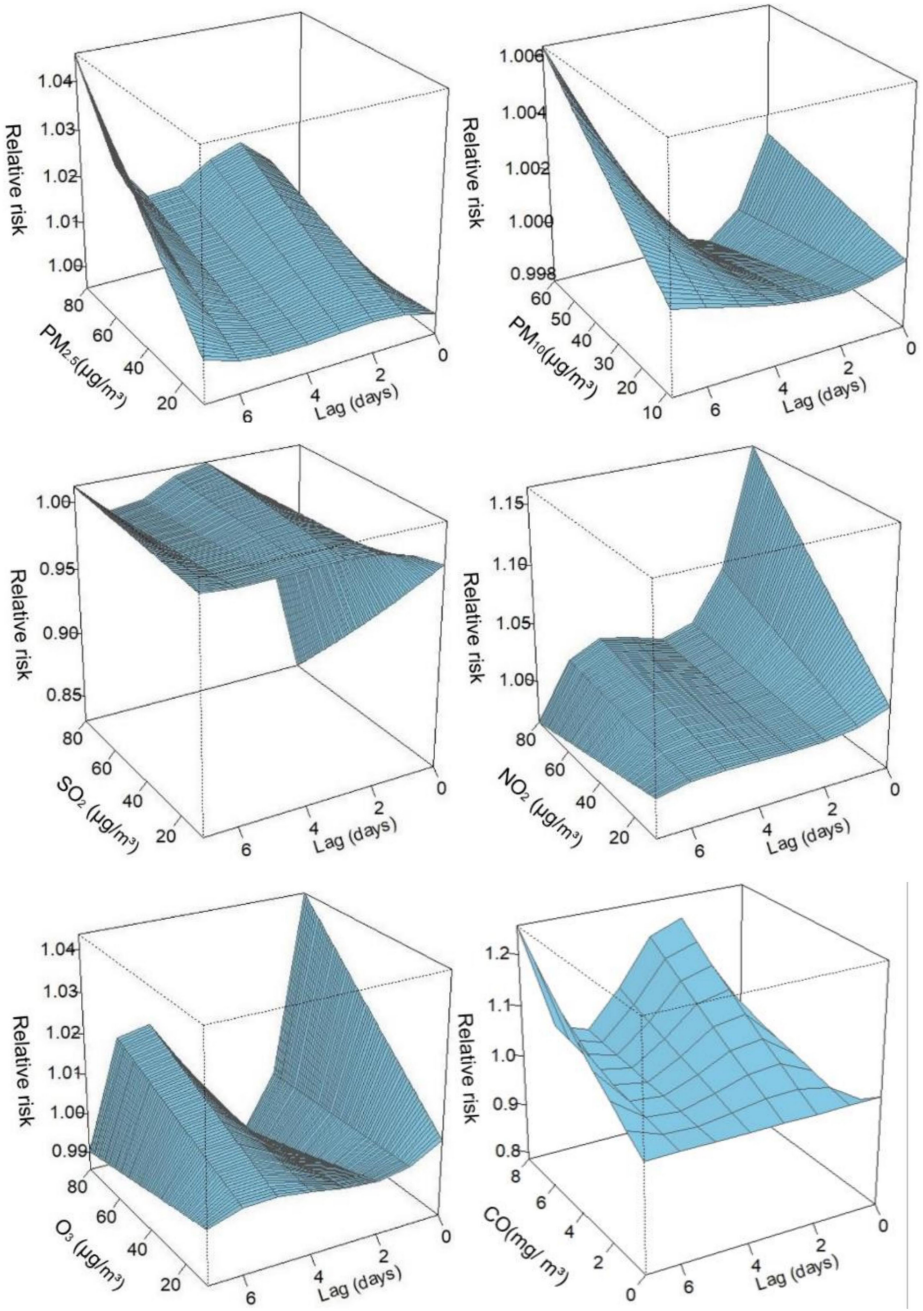
Three-dimensional graphs. PM_2.5_, aerodynamic diameter<2.5 μm; PM_10_, aerodynamic diameter<10 μm; SO_2_, sulfur dioxide; NO_2_, nitrogen dioxide; CO, carbon monoxide; O_3_, ozone.

The relationship between pollutants and the number of diabetes hospitalizations showed a largely nonlinear and lagged relationship. Three-dimensional (3D) plots were used to describe the relationship between the six air pollutants and the number of hospitalizations for diabetes (**Figure 2**). The 3D graphic represents the correlation in terms of RR, depicting the risk of hospitalization for diabetes mellitus and the exposure to different pollutants at different lag days.

**Table 2** shows the RR when exposed to the six air pollutants at different lag days, including single-day and cumulative lags. The single-day lag RR of PM_2.5_ was detected as significant at lag 1–lag 7. The cumulative lag RR for PM_2.5_ exhibited the greatest effect at lag 07 (RR=1.031; 95% CI: 1.007–1.056). Similarly, the effect of NO_2_ on the single-day lag RR was significant from lag 0–lag 6, and the strongest association of cumulative lag occurred at lag 06 (RR=1.067; 95% CI: 1.016–1.121). The single-day lag RR of CO was significant at lag 1–lag 7, with the highest effect of cumulative lag at lag 07 (RR=1.139; 95% CI: 0.964–1.345). Regarding O_3_, adverse effects of the single-day lag occurred at lag 4–lag 6, and the greatest cumulative effect estimation was observed at lag 06 (RR=1.013; 95% CI: 0.992–1.036). However, no correlation was observed between PM_10_ and SO_2_ and the risk of hospitalization for diabetes.

**Table 2:** Relative risk (RR) (95% confidence intervals [CIs]) of diabetes admissions with an increase of 10 μg/m^3^ in air pollutants (and 1 mg/m^3^ in carbon monoxide [CO]).

Lag days	PM_2.5_	PM_10_	SO_2_	NO_2_	O_3_	CO
	RR (95% CI)	RR (95% CI)	RR (95% CI)	RR (95% CI)	RR (95% CI)	RR (95% CI)
**Lag 0**	0.999(0.987–1.011)	1.000(0.994–1.006)	0.959(0.915–1.004)	1.034(1.006–1.063)	1.009(0.997–1.022)	0.947(0.866–1.035)
**Lag 1**	1.003(0.998–1.008)	0.999(0.996–1.003)	0.985(0.963–1.007)	1.013(0.999–1.027)	0.999(0.993–1.006)	1.017(0.977–1.058)
**Lag 2**	1.004(0.999–1.010)	0.999(0.996–1.003)	0.998(0.974–1.023)	1.004(0.988–1.019)	0.996(0.990–1.003)	1.043(0.998–1.090)
**Lag 3**	1.004(0.999–1.008)	0.999(0.996–1.002)	1.002(0.983–1.022)	1.002(0.990–1.014)	0.998(0.993–1.003)	1.038(1.003–1.075)
**Lag 4**	1.003(0.998–1.007)	0.999(0.997–1.003)	1.001(0.983–1.019)	1.004(0.992–1.016)	1.001(0.996–1.006)	1.020(0.985–1.056)
**Lag 5**	1.003(0.997–1.009)	1.000(0.997–1.004)	0.998(0.975–1.022)	1.005(0.990–1.021)	1.004(0.998–1.010)	1.005(0.960–1.051)
**Lag 6**	1.005(0.999–1.010)	1.001(0.998–1.004)	0.997(0.977–1.018)	1.002(0.989–1.0157)	1.004(0.998–1.009)	1.010(0.972–1.049)
**Lag 7**	1.010(1.000–1.020)	1.002(0.996–1.008)	1.002(0.963–1.043)	0.991(0.966–1.0176)	0.997(0.987–1.008)	1.052(0.974–1.135)
**Lag 01**	1.002(0.988–1.017)	1.000(0.992–1.008)	0.944(0.892–0.999)	1.048(1.013–1.084)	1.009(0.993–1.026)	0.963(0.865–1.073)
**Lag 02**	1.006(0.991–1.022)	1.000(0.991–1.008)	0.942(0.886–1.002)	1.052(1.015–1.091)	1.006(0.988–1.024)	1.005(0.898–1.126)
**Lag 03**	1.010(0.994–1.027)	0.999(0.989–1.008)	0.945(0.883–1.010)	1.055(1.014–1.096)	1.004(0.985–1.023)	1.044(0.926–1.178)
**Lag 04**	1.013(0.996–1.031)	0.999(0.989–1.008)	0.946(0.879–1.017)	1.059(1.016–1.103)	1.005(0.986–1.025)	1.066(0.937–1.212)
**Lag 05**	1.016(0.996–1.036)	0.999(0.988–1.010)	0.944(0.871–1.023)	1.064(1.018–1.113)	1.009(0.989–1.031)	1.071(0.928–1.237)
**Lag 06**	1.021(0.998–1.044)	1.000(0.988–1.013)	0.942(0.862–1.029)	1.067(1.016–1.121)	1.013(0.992–1.036)	1.082(0.922–1.270)
**Lag 07**	1.031(1.007–1.056)	1.002(0.989–1.016)	0.944(0.859–1.037)	1.058(1.005–1.114)	1.011(0.989–1.034)	1.139(0.964–1.345)

lag0, zero day lag; lag7, seventh day lag; lag01, one day cumulative lag; lag07, seven days cumulative lag; PM2.5, Aerodynamic diameter<2.5 mm; PM_10_, Aerodynamic diameter<10 mm; SO_2_, sulphur dioxide; NO_2_, nitrogen dioxide; CO, carbon monoxide; O_3_, ozone; RR, relative risk; CI, confidence interval.

### 3.4 Association between pollutants, length of hospital stay, and hospital cost

Hospitalization costs and LOS varied among patients with diabetes under the influence of different pollutants, as quantified by the AF and attribution number (**Table 3**). Among the six pollutants, CO had the highest AF, reaching 10.8% and 10.41%, respectively, which resulted in an additional hospital cost of 10.41 million Chinese Yuan and 132,500 hospitalization days (**Table 3**). The AF of NO_2_ was also high, reaching 5.4% and 4.1%, respectively, leading to an increase of 5.2 million Chinese Yuan in hospital costs and 710,100 hospitalization days. In addition, PM_2.5_, PM_10_, and O_3_ were significantly associated with LOS and hospital costs, while SO_2_ was negatively correlated with hospital LOS and costs. **Supplementary Tables S1 and S2** show the single-day lag RR and cumulative lag RR of hospital cost, LOS, and their CIs for the six different pollutants on varying lag days.

**Table 3:** Attributable fraction and attributable number of hospital cost and length of stay of patients with diabetes.

Pollution	Hospital cost		LOS	
	AF (%)	AN (million)	AF (%)	AN (hundred)
**PM_2.5_**	4.80(1.70–7.70)	4.62(1.63–7.42)	3.70(1.38–6.10)	64.08(23.90–105.66)
**PM_10_**	1.50(-0.20–3.20)	1.44(-0.19–3.08)	0.40(-0.90–1.80)	6.92(-15.58–31.17)
**SO_2_**	-4.40(-18.90–8.10)	-4.24(-18.22–7.81)	-8.50(-19.90–1.50)	-14,723(-344.69–25.98)
**NO_2_**	5.40(-1.10–11.60)	5.20(-1.06–11.18)	4.10(-1.20–9.20)	71.01(-20.78–159.35)
**O_3_**	1.90(-1.00–4.80)	1.83(-0.96–4.62)	1.10(-1.20–3.40)	19.05(-20.78–58.89)
**CO**	10.80(-4.00–33.90)	10.41(-3.80–32.68)	13.40(-3.00–27.30)	132.5 (-51.96–472.87)

AF, attribution fraction; AN, attribution number; LOS, length of stay; PM_2.5_, aerodynamic diameter<2.5 μm; PM_10_, aerodynamic diameter<10 μm; SO_2_, sulphur dioxide; NO_2_, nitrogen dioxide; CO, carbon monoxide; O_3_, ozone.

### 3.5 Subgroup analysis

**Figure 3:**
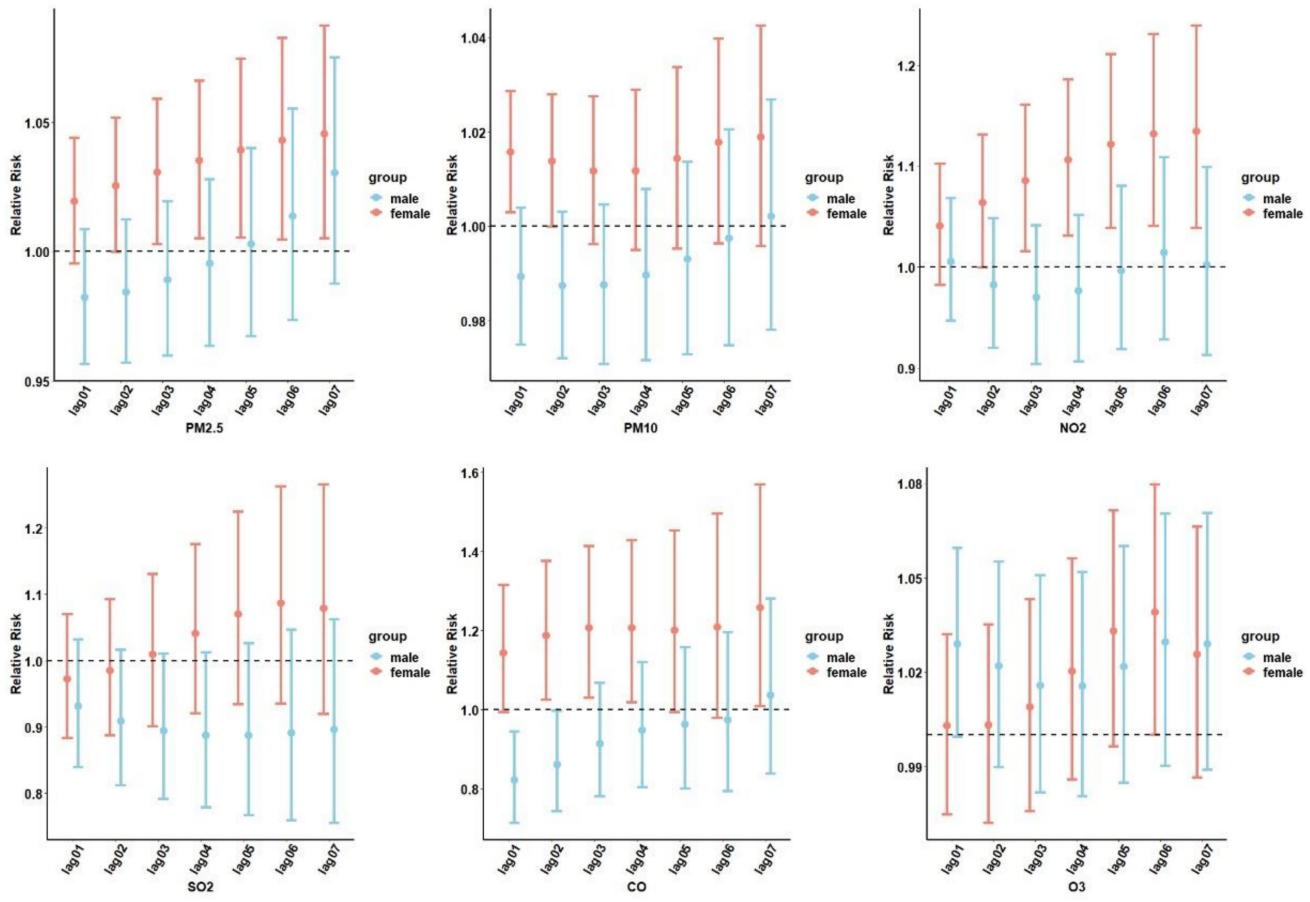
The cumulative lag relative risk (RR) (95% confidence interval [CI]) of hospital admissions for diabetes stratified by sex. PM_2.5_, aerodynamic diameter<2.5 μm; PM_10_, aerodynamic diameter<10 μm; SO_2_, sulfur dioxide; NO_2_, nitrogen dioxide; CO, carbon monoxide; O_3_, ozone.

The cumulative lagged RR of air pollution-related diabetes by sex stratification is depicted in **Figure 3**. The six air pollutants showed differences under the sex stratification, with different values for the associated RRs: PM_2.5_ at lag 07 (RR=1.045; 95% CI: 1.005–1.087), PM_10_ at lag07 (RR=1.018; 95% CI: 0.995–1.042), SO_2_ at lag 06 (RR=1.086; 95% CI: 0.935–1.262), NO_2_ at lag 07 (RR=1.134; 95% CI: 1.038–1.239), and CO at lag 07 (RR=1.258; 95% CI: 1.008–1.159). No significant associations were observed between O_3_, diabetes admissions, and sex.

**Figure 4:**
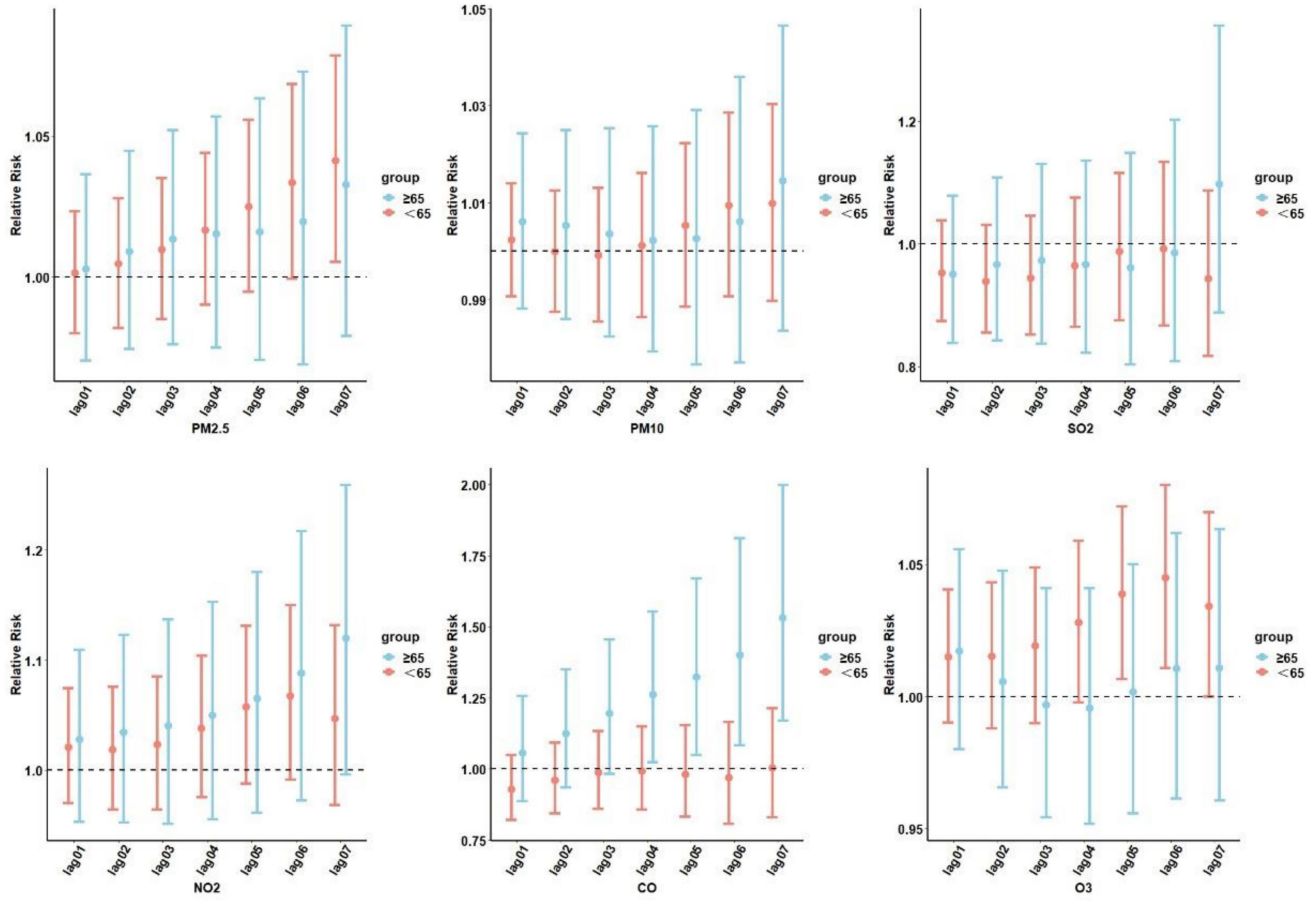
Cumulative lag RR with 95% CIs of diabetes-related hospital admissions associated with air pollutants at various lags stratified by age. PM_2.5_, aerodynamic diameter<2.5 μm; PM_10_, aerodynamic diameter<10 μm; SO_2_, sulfur dioxide; NO_2_, nitrogen dioxide; CO, carbon monoxide; O_3_, ozone.

We performed an analysis of the correlation between air pollution and diabetes under different age stratifications (**Figure 4**). People<65 years of age were more likely to be affected by O_3_ exposure and develop T2DM.

Those who were ≥65 years old and were exposed to NO_2_ and CO were more likely to develop T2DM. At lag06, exposure to O_3_ was significantly associated with diabetes admissions for individuals<65 years of age (RR=1.044; 95% CI: 1.010–1.080). For the ≥65-years-old subgroups, the most significant association between CO and NO_2_ and diabetes hospitalization was observed on the sixth day, with NO_2_ (RR=1.087; 95% CI: 0.972–1.216) and CO (RR=1.529; 95% CI: 1.170–1.997). No age-related risk of diabetes was observed with PM_2.5_, PM_10_, and SO_2_ exposure.

Significant differences in diabetes effect estimates were found under the warm and cold season stratification (**Table 4**). The effect estimates of three significant pollutants, SO_2_, O_3,_ and CO, had greater effect values in the cold season than in the warm season. The highest RRs (95% CI) for SO_2_, O_3_, and CO on diabetes hospital admission were observed at lag 07 with 1.059 (95% CI: 0.909–1.235), at lag 07 with 1.060 (95% CI: 0.961–1.170), and at lag 07 with 1.121 (95% CI: 0.859–1.463), respectively. However, PM_2.5_, PM_10,_ and NO_2_ did not significantly affect hospital admissions across seasons.

**Table 4:** RR (95% CIs) of diabetes admissions with an increase of 10 μg/m^3^ in air pollutants (and 1 mg/m^3^ in CO) according to the single-pollutant model by season.

Lag days		PM_2.5_	PM_10_	SO_2_	NO_2_	O_3_	CO
		RR (95% CI)	RR (95% CI)	RR (95% CI)	RR (95% CI)	RR (95% CI)	RR (95% CI)
**Cold**	**Lag 0**	0.993(0.977–1.009)	0.991(0.980–1.002)	0.967(0.904–1.034)	1.008(0.964–1.053)	0.997(0.959–1.036)	0.938(0.834–1.055)
	**Lag 1**	1.003(0.996–1.010)	1.000(0.995–1.005)	1.009(0.977–1.041)	0.999(0.978–1.020)	1.003(0.982–1.025)	1.022(0.969–1.078)
	**Lag 2**	1.006(0.999–1.013)	1.003(0.997–1.008)	1.021(0.987–1.055)	0.993(0.971–1.016)	1.004(0.983–1.025)	1.047(0.992–1.106)
	**Lag 3**	1.005(0.999–1.010)	1.002(0.998–1.006)	1.014(0.989–1.040)	0.989(0.972–1.007)	1.002(0.985–1.019)	1.033(0.989–1.078)
	**Lag 4**	1.002(0.996–1.007)	0.999(0.995–1.003)	1.001(0.977–1.026)	0.987(0.970–1.004)	1.000(0.983–1.017)	1.005(0.962–1.048)
	**Lag 5**	0.999(0.992–1.007)	0.998(0.993–1.003)	0.994(0.962–1.027)	0.986(0.965–1.008)	1.003(0.983–1.024)	0.987(0.934–1.043)
	**Lag 6**	1.002(0.995–1.008)	0.999(0.995–1.004)	1.004(0.975–1.034)	0.987(0.968–1.006)	1.013(0.994–1.032)	1.004(0.955–1.055)
	**Lag 7**	1.011(0.998–1.023)	1.007(0.997–1.016)	1.045(0.991–1.103)	0.989(0.953–1.027)	1.034(0.999–1.070)	1.082(0.985–1.198)
	**Lag 01**	0.996(0.976–1.017)	0.991(0.977–1.005)	0.976(0.897–1.062)	1.007(0.954–1.064)	1.000(0.949–1.054)	0.960(0.827–1.113)
	**Lag 02**	1.003(0.981–1.025)	0.994(0.979–1.010)	0.997(0.908–1.094)	1.001(0.943–1.062)	1.005(0.946–1.067)	1.005(0.857–1.180)
	**Lag 03**	1.007(0.984–1.032)	0.997(0.980–1.013)	1.012(0.914–1.120)	0.990(0.929–1.055)	1.007(0.941–1.077)	1.039(0.874–1.234)
	**Lag 04**	1.009(0.983–1.036)	0.997(0.979–1.015)	1.013(0.908–1.131)	0.977(0.914–1.045)	1.008(0.936–1.085)	1.044(0.866–1.259)
	**Lag 05**	1.009(0.980–1.039)	0.995(0.975–1.015)	1.008(0.891–1.140)	0.965(0.896–1.038)	1.011(0.932–1.097)	1.031(0.834–1.274)
	**Lag 06**	1.011(0.978–1.046)	0.995(0.972–1.018)	1.013(0.881–1.164)	0.953(0.878–1.034)	1.025(0.937–1.121)	1.036(0.813–1.318)
	**Lag 07**	1.022(0.985–1.061)	1.002(0.977–1.028)	1.059(0.909–1.235)	0.943(0.862–1.032)	1.060(0.961–1.170)	1.121(0.859–1.463)
**Warm**	**Lag 0**	1.022(0.977–1.070)	1.012(0.990–1.033)	0.935(0.840–1.040)	1.003(0.943–1.067)	1.015(0.996–1.034)	0.904(0.715–1.143)
	**Lag 1**	1.006(0.982–1.030)	1.003(0.992–1.014)	0.956(0.907–1.008)	0.993(0.960–1.028)	1.001(0.992–1.010)	0.965(0.856–1.089)
	**Lag 2**	1.000(0.975–1.025)	0.999(0.988–1.010)	0.986(0.932–1.042)	0.993(0.959–1.029)	0.997(0.988–1.005)	1.008(0.881–1.153)
	**Lag 3**	1.000(0.979–1.021)	0.998(0.989–1.007)	1.014(0.969–1.061)	0.999(0.970–1.028)	0.998(0.992–1.005)	1.031(0.924–1.150)
	**Lag 4**	1.003(0.982–1.024)	0.998(0.989–1.007)	1.031(0.987–1.078)	1.005(0.976–1.035)	1.002(0.996–1.009)	1.035(0.929–1.153)
	**Lag 5**	1.004(0.979–1.030)	0.999(0.987–1.010)	1.028(0.972–1.087)	1.008(0.973–1.044)	1.005(0.996–1.013)	1.022(0.894–1.168)
	**Lag 6**	1.000(0.976–1.024)	0.997(0.986–1.007)	0.994(0.945–1.047)	1.003(0.971–1.036)	1.001(0.994–1.008)	0.994(0.882–1.120)
	**Lag 7**	0.986(0.946–1.028)	0.991(0.972–1.011)	0.925(0.841–1.018)	0.987(0.931–1.047)	0.989(0.975–1.002)	0.955(0.764–1.194)
	**Lag 01**	1.029(0.96–1.093)	1.015(0.988–1.043)	0.894(0.781–1.025)	0.997(0.918–1.082)	1.016(0.991–1.042)	0.873(0.653–1.169)
	**Lag 02**	1.029(0.961–1.102)	1.014(0.984–1.045)	0.882(0.759–1.025)	0.991(0.900–1.091)	1.013(0.986–1.041)	0.881(0.637–1.217)
	**Lag 03**	1.030(0.954–1.111)	1.012(0.980–1.046)	0.895(0.758–1.056)	0.990(0.887–1.104)	1.012(0.984–1.042)	0.908(0.634–1.301)
	**Lag 04**	1.033(0.951–1.123)	1.011(0.977–1.047)	0.923(0.771–1.106)	0.995(0.883–1.122)	1.015(0.986–1.045)	0.940(0.638–1.386)
	**Lag 05**	1.038(0.946–1.138)	1.010(0.973–1.049)	0.950(0.776–1.162)	1.003(0.878–1.146)	1.020(0.989–1.053)	0.961(0.624–1.479)
	**Lag 06**	1.038(0.937–1.149)	1.007(0.966–1.051)	0.945(0.754–1.184)	1.007(0.870–1.166)	1.022(0.988–1.057)	0.956(0.591–1.548)
	**Lag 07**	1.024(0.915–1.145)	0.999(0.954–1.047)	0.875(0.684–1.119)	0.995(0.848–1.167)	1.011(0.976–1.047)	0.914(0.539–1.549)

cold, cold season; warm, warm season; lag0:lag0, zero day lag; lag7, seventh day lag; lag01, one day cumulative lag; lag07, seven days cumulative lag; PM_2.5_, Aerodynamic diameter<2.5 mm; PM_10_, Aerodynamic diameter<10 mm; SO2, sulphur dioxide; NO_2_, nitrogen dioxide; CO, carbon monoxide; O_3_, ozone; RR, relative risk; CI, confidence interval.

**Figure 5:**
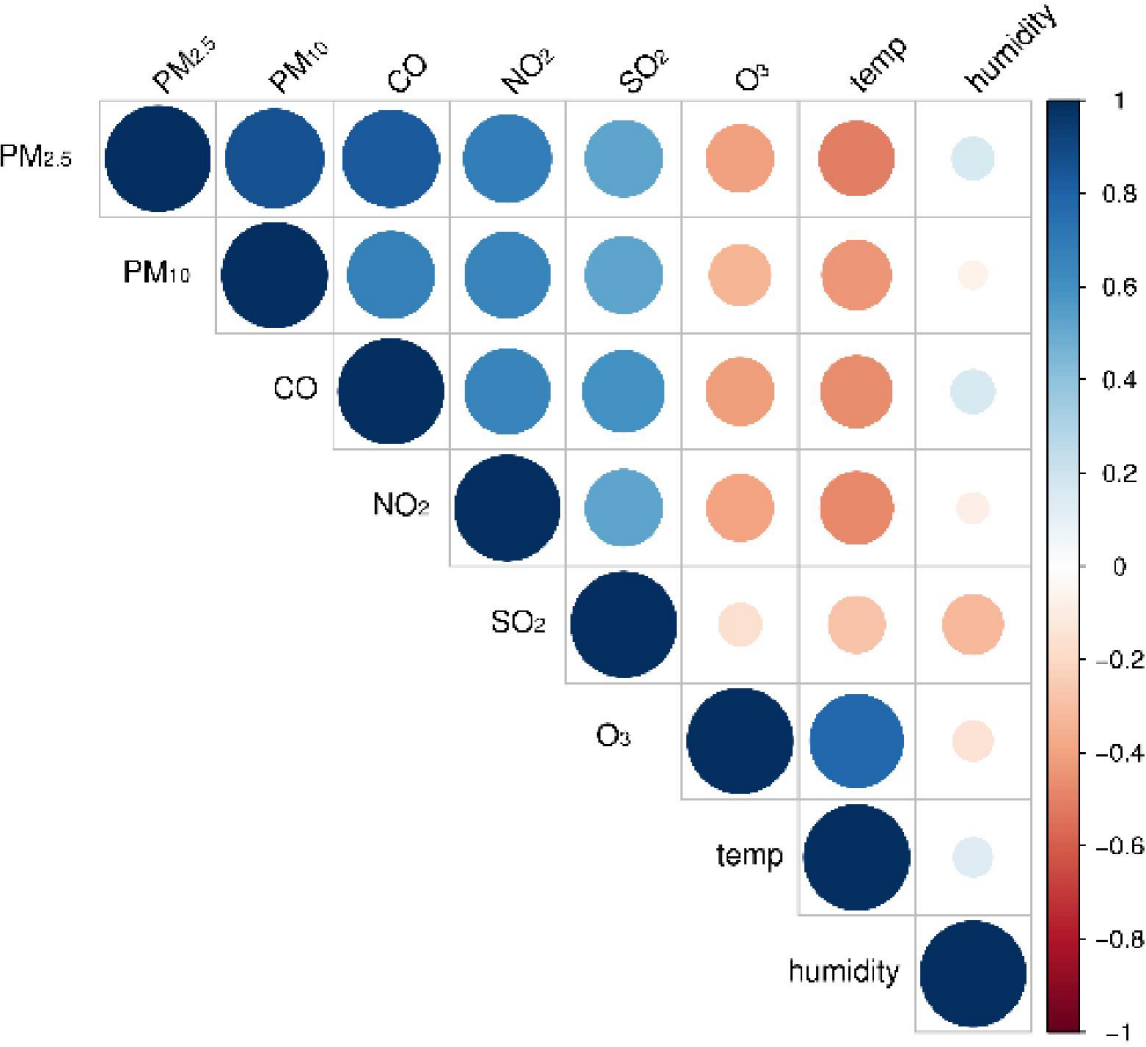
Spearman’s correlation analysis between air pollutants and meteorological variables. PM_2.5_, aerodynamic diameter<2.5 μm; PM_10_, aerodynamic diameter<10 μm; SO_2_, sulfur dioxide; NO_2_, nitrogen dioxide; CO, carbon monoxide; O_3_, ozone.

**Figure 5** presents the Spearman’s correlation analysis results between air pollutants and meteorological factors. There was a positive correlation between particulate pollutants and NO_2_, SO_2_, and CO, with fluctuating correlation coefficients of 0.48–0.87, and O_3_ was negatively associated with other air pollutants and humidity. Temperature was negatively correlated with all air pollutants except O_3_. Apart from PM_2.5_, CO, and temperature, all pollutants were negatively correlated with humidity.

When analyzing a single air pollutant, each 10 μg/m^3^ increase in the concentration of particulate pollutants, NO_2_, and O_3_ increased the risk of diabetes by 3.1%, 0.2%, 5.8%, and 1.1%, respectively. The risk of hospitalization for diabetes increased by 13.9% for every 1 mg/m^3^ increase in CO concentration. In the double-pollution model, the RR of O_3_ adjusted for the other five air pollutants were all smaller than the single exposure RR of O_3_, which corresponded to the Spearman’s correlation coefficient. **Table 5** demonstrates the risk of diabetes in the single-pollutant exposure and dual-pollutant models for each 10 μg/m^3^ (1 mg/m^3^) increase in air pollutant concentration. The trend of the double-pollution model aligned with the Spearman’s correlation coefficient, demonstrating the robustness of the impact estimates of the six pollutants.

**Table 5:** Fitting results of single and double air pollutant model.

Model	RR (95% CI)	Model	RR (95% CI)
**PM_2.5_**	1.031(1.007–1.055)	**SO_2_**	0.944 (0.859–1.037)
**PM_2.5_+PM_10_**	1.040(0.999–1.081)	**SO_2_+PM_2.5_**	0.926 (0.835–1.028)
**PM_2.5_+NO_2_**	1.010(0.979–1.041)	**SO_2_+PM_10_**	0.919 (0.829–1.018)
**PM_2.5_+SO_2_**	1.029(1.003–1.055)	**SO_2_+NO_2_**	0.838 (0.748–0.938)
**PM_2.5_+CO**	1.027(0.994–1.060)	**SO_2_+CO**	0.957 (0.862–1.061)
**PM_2.5_+O_3_**	1.025(1.000–1.051)	**SO_2_+O_3_**	0.935 (0.846–1.035)
**PM_10_**	1.002(0.989–1.015)	**CO**	1.139 (0.964–1.345)
**PM_10_+PM_2.5_**	0.985(0.964–1.008)	**CO+PM_2.5_**	0.983 (0.782–1.236)
**PM_10_+NO_2_**	0.996(0.979–1.013)	**CO+PM_10_**	1.121 (0.925–1.359)
**PM_10_+SO_2_**	1.007(0.992–1.022)	**CO+NO_2_**	1.012 (0.832–1.232)
**PM_10_+CO**	0.999(0.984–1.015)	**CO+SO_2_**	1.140 (0.955–1.360)
**PM_10_+O_3_**	1.005(0.991–1.019)	**CO+O_3_**	1.123 (0.944–1.337)
**NO_2_**	1.058(1.005–1.113)	**O_3_**	1.011 (0.989–1.034)
**NO_2_+PM_2.5_**	1.065(0.999–1.136)	**O_3_+PM_2.5_**	0.994 (0.971–1.019)
**NO_2_+PM_10_**	1.081(1.015–1.151)	**O_3_+PM_10_**	0.995 (0.971–1.019)
**NO_2_+SO_2_**	1.118(1.053–1.188)	**O_3_+NO_2_**	0.994 (0.970–1.018)
**NO_2_+O_3_**	1.075(1.019–1.134)	**O_3_+SO_2_**	0.995 (0.971–1.020)
**NO_2_+CO**	1.077(1.015–1.143)	**O_3_+CO**	1.001 (0.977–1.025)

RR, relative risk; CI, confidence interval; PM_2.5_, aerodynamic diameter<2.5 μm; PM_10_, aerodynamic diameter<10 μm; SO_2_, sulphur dioxide; NO_2_, nitrogen dioxide; CO, carbon monoxide; O_3_, ozone.

To verify the robustness of the model, a sensitivity analysis was performed. The output values of the six different pollutants for 7 days of cumulative lag were calculated after making changes in the degrees of freedom for lag days and temperature. It was found that the output values of RR and 95% CIs of air pollution and T2DM hospitalization correlations at different degrees of freedom were within a relatively stable range (**Supplementary Table S3**).

## 4 Discussion

The DLNM was used to clarify the relationship between air pollution and diabetes admissions, LOS, and hospitalization expenses with respect to variables and lagged days. The findings indicated that PM_2.5_, NO_2_, CO, and O_3_ were positively correlated with T2DM hospitalizations, with varying effects across different lag days and subgroups. In the single-pollution model, we found that each ten-unit increase in PM_2.5_, NO_2_, and O_3_ and each unit increase in CO was significantly associated with diabetes, with RR values of 1.031 (1.007–1.056), 1.058 (1.005–1.114), 1.011 (0.989–1.034), and 1.139 (0.964–1.345), respectively. Similarly, a previous study found that PM_2.5_ and NO_2_ was positively associated with the risk of death due to diabetes in the United States, whereas no association was observed for O_3_ (28, 29). Song et al. (29) demonstrated that each ten-unit increase in PM_2.5_, PM_10_, SO_2_, and NO_2_ and each unit increase in CO corresponded to an increase in T2DM hospitalization. Moreover, Paul et al. (30) observed significant associations between T2DM and PM_2.5_ and O_3_. However, our study depicted a limited relationship between PM_10_ levels and the prevalence of diabetes, contrasting with the results of most previous studies. Thus, further research is required to confirm these findings.

There is limited research on the correlation between air pollution and hospital costs and LOS due to diabetes mellitus. We observed that air pollutants other than SO_2_ were positively correlated with LOS and hospital costs for patients with diabetes, resulting in a significant economic burden. Notably, neglecting the importance of health during development results in higher future costs (31). Therefore, the relevant authorities and medical institutions should pay attention to the impact of air pollution and take appropriate measures to reduce the economic costs and health impacts of air pollution.

The seasonal subgroup analysis depicted correlations between diabetes prevalence and PM_10_, SO_2_, O_3_, and CO during the cold season. Studies have demonstrated that older adults and individuals in cold seasons are more susceptible to outdoor air pollutants (32). Air pollution arises from a combination of anthropogenic emissions and meteorological factors (33, 34). Focusing solely on the individual effects of pollutants or meteorological factors may lead to an oversight of the overall health effects of the mixture. Air pollutants impact both health and climate; seasonal and regional differences may change the concentration of particulate matter, consequently affecting the correlation between air pollution and coronary heart disease (35). Seasonal transitions (from winter to summer) and gradually improving meteorological factors help reduce the impact of anthropogenic emissions on air pollution (36).

The sex-stratified subgroup analysis revealed that females were more susceptible to air pollution in terms of developing T2DM than males were. This may be attributed to the distinct physiological structure of females. Air pollution affects females more, possibly because of sex-related biological differences, such as hormones, body shape, diet, and activity patterns (37). Furthermore, the airway diameters in females are different to those in males, facilitating the deposition of PM_2.5_ particles (38).

In the age-stratified analysis, the effect of air pollution in younger people was slightly lower than that in individuals aged >65 years. This was mainly observed with NO_2_ and CO. Abnormal glucose metabolism caused by air pollution in older adults may have led to this result (39). Therefore, air pollution may be a risk factor for diabetes in older individuals. In contrast, O_3_ exhibited the opposite effect, potentially owing to differences in outdoor activity time, with the increased engagement of younger people in outdoor activities and thus increased inhalation of air pollutants potentially explaining the stronger effect of air pollutants on this group (40). More walking and higher levels of greenery are negatively related with T2DM (7). However, previous research by Kim et al. showed that older people exposed to air pollution have a lower risk of diabetes during moderate- and high-intensity physical activities (41). Furthermore, studies have demonstrated that healthy physical activity habits can reduce the risk of T2DM, even in the presence of PM_2.5_ exposure (42). However, further research is required to validate this assertion.

**Figure 6:**
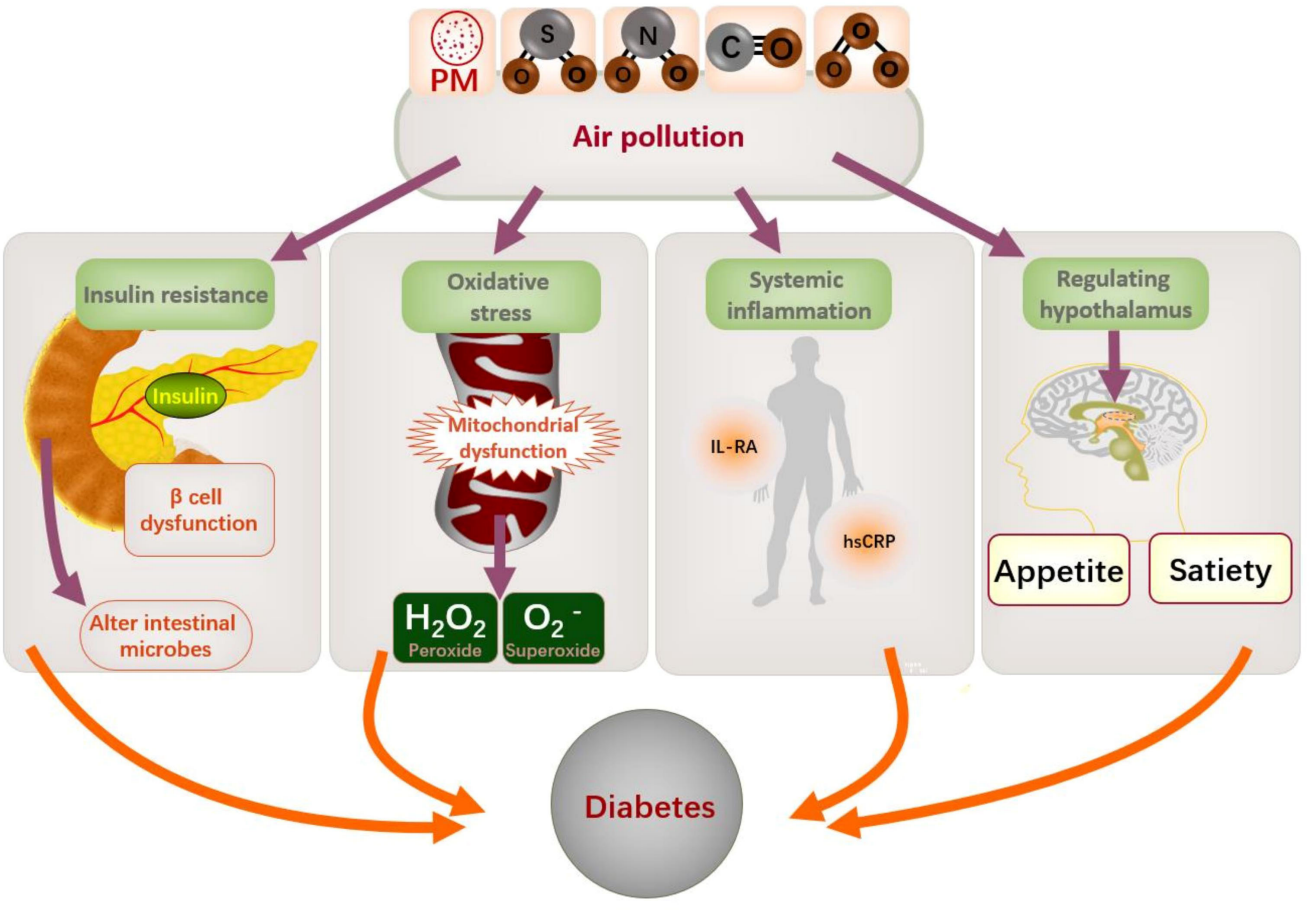
Mechanism of air pollution and diabetes. PM, particulate matter; IL-RA, interleukin receptor antagonists; hsCRP: high-sensitivity C-reactive protein.

Air pollution could constitute a risk factor for T2DM development (16). There are various pathophysiologic mechanisms by which air pollution may contribute to diabetes. Previous studies have identified potential pathological pathways involved in air pollution, including insulin resistance (43–45), β-cell dysfunction, neurohormonal dysfunction (46), endothelial dysfunction (47), systemic inflammation, and alterations in the composition and diversity of gut microbiota (48). Greater exposure to air pollution is associated with increased circulating levels of adiponectin, interleukin-1 receptor antagonists, and high-sensitivity C-reactive protein (49). Hypothesized effects of air pollutants encompass impaired endothelial function, elevated systemic inflammation, mitochondrial dysfunction, and oxidative stress, which may contribute to the development of T2DM (50). Furthermore, air pollutants may modulate inflammatory responses in the homeostatic centers of the brain and may modulate hypothalamic mechanisms that regulate appetite and satiety (51). In addition, prolonged exposure to PM_10_ and NO_2_ pollution is positively related with glycated hemoglobin (52), and PM_2.5_ pollution has resulted in a massive T2DM burden worldwide (53). **Figure 6** shows the mechanisms underlying the association between air pollution and T2DM.

It is well known that wearing a mask can effectively avoid exposure to air pollution. Antioxidants also play an important role in avoiding the damage caused by air pollution. A diet that promotes oxidation and inflammation will strengthen the harmful effects of air pollutants on the body (54), while a healthy diet, adhering to the Mediterranean diet and taking antioxidants from fruits and vegetables can reduce the burden of diseases caused by air pollution (54, 55). A healthy diet can effectively prevent T2DM (56). Adequate intake of antioxidants such as vitamins C and E through diet can reduce diabetes caused by air pollution (57). In addition to the common air pollution such as particulate matter, toxic environmental substances also endanger our health, such as poisons caused by gasoline tail gas. Studies have shown that the intervention of antioxidant vitamin E can restore the airway injury of rats exposed to poisons (58). Therefore, intake of antioxidants is beneficial to health.

In Pr Jean-Jacques Laffont’s book *The Economics of Uncertainty and Information*, the influence of taxation on agents inspires us that increasing the taxation of polluting enterprises may reduce the willingness of enterprises to invest, which is not conducive to economic development. Conversely, if the government introduces policies to improve the air pollution caused by factories and other enterprises, and gives tax relief to some environmental protection enterprises, air pollution may be further prevented.

This study had some limitations. First, the patients with diabetes in this study did not include all patients in Xinxiang, potentially resulting in the oversight of individuals with insignificant clinical symptoms and leading to underestimation. Second, this study analyzed data collected over 5 years, possibly compromising the model’s stability. Third, this study was observational and ecological, preventing us from making causal inferences, despite our efforts to reduce confounding bias through various methods. This study also had several strengths. First, we used the DLNM to more accurately reflect the lagged relationship between air pollution and disease. Second, we measured several aspects of the correlation between air pollution and T2DM, including hospital admissions, LOS, and hospital costs, which may lead to a greater understanding of the economic impact and social pressures of the disease.

In conclusion, air pollutants were positively related with hospitalization rates, expenses, and LOS in patients with diabetes, particularly PM_2.5_, NO_2_, O_3_, and CO. This association was also affected by sex, age, and season. These findings may guide efforts to prevent T2DM and highlight the multiple benefits of improving the air quality in Xinxiang and other highly polluted regions.

## Data Availability

The original contributions presented in the study are included in the article/**Supplementary Material**. Further inquiries can be directed to the corresponding author.
