# Supplementary material for: Exposure to air pollution and the risk of type II diabetes mellitus: a time-series study
Source: Front Endocrinol (Lausanne). 2024 Dec 3;15:1482063. doi: 10.3389/fendo.2024.1482063 (PMC11653192; doi:10.3389/fendo.2024.1482063)
Supplement: Supplementary file 1 [file Table1.docx]

**Supplementary material**

**Table 1S.** **Relative risk (RR) (95% confidence intervals [CIs]) of length of stay with an increase of 10μg/m^3^ in air pollutants**

**(and 1 mg/m^3^ in carbonic oxide [CO]) according to single-pollutant model**

| **Lag days** | **PM_2.5_ (95% CI)** | | **PM_10_ (95% CI)** | | **SO_2_ (95% CI)** | | **NO_2_ (95% CI)** | | **O_3_ (95%**  **CI)** | | **CO (95% CI)** | |
| --- | --- | --- | --- | --- | --- | --- | --- | --- | --- | --- | --- | --- |
| **Lag 0** | 1.002(0.986–1.018) | 1.004(0.995–1.012) | | 0.957(0.898–1.021) | | 1.024(0.987–1.063) | | 1.012(0.995–1.030) | | 0.984(0.872–1.110) | |  |
| **Lag 1** | 1.006(0.999–1.013) | 1.001(0.997–1.005) | | 0.990(0.960–1.021) | | 1.012(0.993–1.030) | | 1.002(0.993–1.010) | | 1.047(0.991–1.105) | |  |
| **Lag 2** | 1.006(0.999–1.014) | 1.000(0.995–1.004) | | 1.005(0.972–1.040) | | 1.005(0.984–1.026) | | 0.998(0.990–1.007) | | 1.057(0.994–1.123) | |  |
| **Lag 3** | 1.005(0.999–1.011) | 0.999(0.995–1.003) | | 1.009(0.982–1.036) | | 1.002(0.986–1.018) | | 0.999(0.992–1.006) | | 1.035(0.987–1.085) | |  |
| **Lag 4** | 1.003(0.997–1.009) | 0.999(0.996–1.003) | | 1.004(0.979–1.031) | | 1.002(0.986–1.018) | | 1.002(0.996–1.009) | | 1.004(0.957–1.053) | |  |
| **Lag 5** | 1.003(0.995–1.011) | 1.001(0.996–1.006) | | 0.998(0.965–1.031) | | 1.003(0.982–1.024) | | 1.004(0.996-1.0135) | | 0.986(0.927–1.049) | |  |
| **Lag 6** | 1.006(0.999–1.013) | 1.003(0.999–1.007) | | 0.993(0.965–1.023) | | 1.004(0.986–1.021) | | 1.003(0.996–1.010) | | 1.002(0.951–1.056) | |  |
| **Lag 7** | 1.014(1.001–1.028) | 1.006(0.998–1.014) | | 0.997(0.943–1.054) | | 1.002(0.967–1.038) | | 0.995(0.982–1.010) | | 1.077(0.970–1.195) | |  |
| **Lag 01** | 1.009(0.989–1.028) | 1.005(0.995–1.016) | | 0.948(0.876–1.026) | | 1.037(0.991–1.084) | | 1.014(0.992–1.037) | | 1.030(0.890–1.192) | |  |
| **Lag 02** | 1.015(0.995–1.036) | 1.005(0.994–1.016) | | 0.954(0.875–1.039) | | 1.042(0.993–1.094) | | 1.013(0.989–1.038) | | 1.089(0.934–1.270) | |  |
| **Lag 03** | 1.021(0.999–1.043) | 1.005(0.992–1.017) | | 0.963(0.877–1.057) | | 1.045(0.992–1.100) | | 1.013(0.987–1.039) | | 1.127(0.957–1.328) | |  |
| **Lag 04** | 1.025(1.001–1.049) | 1.005(0.991–1.018) | | 0.967(0.875–1.069) | | 1.047(0.992–1.106) | | 1.016(0.989–1.043) | | 1.132(0.951–1.348) | |  |
| **Lag 05** | 1.028(1.002–1.056) | 1.006(0.991–1.021) | | 0.965(0.865–1.078) | | 1.051(0.991–1.115) | | 1.021(0.993–1.049) | | 1.117(0.919–1.358) | |  |
| **Lag 06** | 1.035(1.005–1.067) | 1.009(0.992–1.026) | | 0.960(0.849–1.084) | | 1.055(0.989–1.126) | | 1.024(0.995–1.055) | | 1.120(0.901–1.393) | |  |
| **Lag 07** | 1.051(1.018–1.084) | 1.016(0.998–1.034) | | 0.957(0.841–1.089) | | 1.058(0.989–1.132) | | 1.020(0.990–1.051) | | 1.206(0.961–1.513) | |  |

PM_2.5_: aerodynamic diameter <2.5 μm; PM_10_: aerodynamic diameter <10 μm; SO_2_: sulphur dioxide; NO_2_: nitrogen dioxide; CO: carbonic oxide; O_3_: ozone; CI: confidence interval

**Table 2S. RR (95% CIs) of hospital cost with an increase of 10μg/m^3^ in air pollutants (and 1 mg/m^3^ in CO) according to single-pollutant model**

| **Lag days** | **PM_2.5_ (95% CI)** | **PM_10_ (95% CI)** | **SO_2_ (95% CI)** | **NO_2_ (95% CI)** | **O_3_ (95% CI)** | **CO (95% CI)** |
| --- | --- | --- | --- | --- | --- | --- |
| **Lag 0** | 0.999(0.986–1.011) | 1.000(0.993–1.006) | 0.942(0.898–0.989) | 1.030(1.000–1.061) | 1.009(0.995–1.023) | 0.943(0.859–1.036) |
| **Lag 1** | 1.005(1.000–1.011) | 1.000(0.997–1.004) | 0.982(0.960–1.005) | 1.011(0.997–1.026) | 0.999(0.992–1.006) | 1.028(0.986–1.072) |
| **Lag 2** | 1.007(1.000–1.013) | 1.000(0.997–1.004) | 1.001(0.976–1.027) | 1.003(0.987–1.019) | 0.996(0.989–1.003) | 1.056(1.007–1.106) |
| **Lag 3** | 1.005(1.000–1.010) | 1.000(0.997–1.003) | 1.006(0.986–1.026) | 1.001(0.988–1.013) | 0.998(0.993–1.003) | 1.044(1.006–1.083) |
| **Lag 4** | 1.003(0.998–1.008) | 0.999(0.996–1.002) | 1.001(0.982–1.021) | 1.001(0.989–1.014) | 1.002(0.996–1.007) | 1.016(0.979–1.055) |
| **Lag 5** | 1.002(0.995–1.008) | 0.999(0.995–1.003) | 0.994(0.970–1.019) | 1.002(0.986–1.019) | 1.004(0.998–1.011) | 0.995(0.949–1.044) |
| **Lag 6** | 1.004(0.999–1.009) | 1.000(0.997–1.004) | 0.991(0.970–1.013) | 1.000(0.986–1.014) | 1.004(0.998–1.010) | 1.003(0.963–1.044) |
| **Lag 7** | 1.012(1.001–1.022) | 1.003(0.996–1.009) | 0.997(0.957–1.039) | 0.991(0.964–1.019) | 0.996(0.985–1.008) | 1.062(0.979–1.151) |
| **Lag 01** | 1.004(0.989–1.020) | 1.000(0.992–1.009) | 0.926(0.872–0.983) | 1.042(1.006–1.080) | 1.008(0.991–1.026) | 0.970(0.866–1.087) |
| **Lag 02** | 1.011(0.995–1.028) | 1.001(0.992–1.010) | 0.928(0.869–0.990) | 1.046(1.006–1.087) | 1.005(0.986–1.024) | 1.025(0.910–1.155) |
| **Lag 03** | 1.017(0.999–1.035) | 1.001(0.992–1.011) | 0.934(0.870–1.002) | 1.047(1.004–1.091) | 1.004(0.983–1.024) | 1.071(0.943–1.216) |
| **Lag 04** | 1.020(1.001–1.039) | 1.001(0.991–1.012) | 0.935(0.867–1.009) | 1.049(1.004–1.095) | 1.006(0.985–1.027) | 1.089(0.951–1.247) |
| **Lag 05** | 1.022(1.001–1.044) | 1.001(0.989–1.013) | 0.931(0.856–1.012) | 1.052(1.003–1.103) | 1.011(0.989–1.033) | 1.084(0.932–1.262) |
| **Lag 06** | 1.027(1.003–1.051) | 1.002(0.988–1.015) | 0.923(0.841–1.013) | 1.052(0.999–1.108) | 1.015(0.992–1.039) | 1.088(0.919–1.288) |
| **Lag 07** | 1.039(1.014–1.065) | 1.005(0.991–1.019) | 0.921(0.834–1.016) | 1.043(0.988–1.102) | 1.012(0.988–1.036) | 1.155(0.970–1.377) |

PM_2.5_: aerodynamic diameter <2.5 μm; PM_10_: aerodynamic diameter <10 μm; SO_2_: sulphur dioxide; NO_2_: nitrogen dioxide; CO: carbonic oxide; O_3_: ozone; CI: confidence interval

**Table 3S RR (95% CI) of output values for six pollutants at different degrees of freedom**

|  | PM_2.5_ | PM_10_ | SO_2_ | NO_2_ | O_3_ | CO |
| --- | --- | --- | --- | --- | --- | --- |
| df=4 | 1.028(1.004,1.052) | 1.004(0.991,1.017) | 0.916(0.836,1.003) | 1.039(0.988,1.092) | 1.000(0.980,1.021) | 1.108(0.941,1.305) |
| df=5 | 1.027(1.004,1.051) | 1.003(0.990,1.017) | 0.915(0.835,1.003) | 1.038(0.987,1.091) | 1.000(0.979,1.021) | 1.108(0.941,1.306) |
| df=6 | 1.027(1.004,1.051) | 1.003(0.990,1.017) | 0.915(0.835,1.003) | 1.037(0.986,1.091) | 1.000(0.979,1.021) | 1.108(0.941,1.306) |

PM_2.5_: aerodynamic diameter <2.5 μm; PM_10_: aerodynamic diameter <10 μm; SO_2_: sulphur dioxide; NO_2_: nitrogen dioxide; CO: carbonic oxide; O_3_: ozone; CI: confidence interval
